# Embedded Cervical Esophagogastrostomy: A Simple and Convenient Method Using a Circular Stapler After Esophagectomy for Esophageal Carcinomas

**DOI:** 10.1245/s10434-013-2991-9

**Published:** 2013-05-05

**Authors:** Sen Wu, Mingyao Chen, Li Wei, Zhong Chen

**Affiliations:** Center of Thoracic Tumor, Henan Provincial People’s Hospital, Zhengzhou, China

## Abstract

**Background:**

Cervical esophagogastrostomy is currently the most common method for esophageal reconstruction after esophagectomy. The advantages and disadvantages of hand-sewn, linear-stapled, or circular-stapled anastomoses have been subject to debate in recent years. We explored a new method of end-to-side anastomosis using a circular stapler that embeds the anastomosis and the remaining esophageal tissue into the gastric cavity to reduce the occurrence of anastomotic leakage and to prevent gastroesophageal reflux.

**Methods:**

In 127 patients with esophageal carcinomas, end-to-side anastomoses with esophageal embedding were performed by connecting the anvil and body of the circular stapler inside the stomach before firing and embedding the anastomosis and remaining esophagus into the stomach after esophagectomy. Retrospective investigations on postoperative complications such as leakage, stricture, and gastroesophageal reflux were conducted.

**Results:**

A total of 123 patients (96.9 %) had successful surgery, and 4 patients (3.3 %) developed anastomotic leakage, with the total morbidity of 20 of 123 (16.3 %) and in-hospital mortality of 1 of 123 (0.8 %). The incidence of stricture (<1 cm) affected 14 of 123 patients (11.4 %). Eight patients underwent dilatation treatment as a result of severe dysphagia (6.5 %). Half of the patients [62 of 123 (50.4 %)] experienced postoperative heartburn, 11 of 123 patients (8.9 %) experienced acid regurgitation, and 16 of 123 patients (13.0 %) experienced nocturnal cough.

**Conclusions:**

Embedded cervical esophagogastrostomy with circular stapler is a simple and convenient method, with low incidence of anastomotic leakage and a good antireflux effect.

Esophageal cancer is the eighth most common malignant tumor worldwide, and surgery is still the preferred method of treatment.^1^ An international survey of 269 surgeons revealed that left cervical esophagogastrostomy is one of the most common procedures used to treat esophageal cancer, and the digestive tract was most frequently restored with a gastric conduit.^2^ Still, esophagogastrostomy remains a challenge for the surgical treatment of esophageal cancer, with common complications that include postoperative anastomotic leakage, anastomotic stricture, and gastroesophageal reflux.^3^,^4^

Since the 1970s, the clinical application of mechanical anastomosis has grown in acceptance. However, the choice of hand-sewn or mechanical and end-to-side or side-to-side techniques of esophagogastrostomy is still under debate. Orringer et al. conducted a study of side-to-side esophagogastrostomy with an EndoGIA stapler (Coviden AG, Dublin, Ireland) in 114 patients and reported only 3 cases of postoperative anastomotic leakage using this method.^5^ A comparative study between side-to-side stapled anastomosis and hand-sewn anastomosis indicated no difference between the methods, suggesting that clinical experience was a more important consideration.^6^ In early studies, circular stapler use was not considered applicable to cervical esophagogastrostomy, although the comparative study of Hsu et al. demonstrated that cervical esophagogastrostomy with circular stapler had similar efficacy as hand-sewn anastomosis.^7^,^8^

At our institution, we perform end-to-side esophagogastrostomy with a circular stapler by embedding the anastomosis and the remaining esophagus into the residual stomach, placing it into the gastric cavity to prevent erosion by saliva, and further surrounding it by the proximal stomach to prevent gastroesophageal reflux. Our study used a retrospective analysis to evaluate the efficacy of this embedded method of cervical esophagogastrostomy with a circular stapler.

## Materials and Methods

### Patient Information

The protocol for this study was reviewed and approved by the institutional review board at the Henan Provincial People’s Hospital, in Zhengzhou, China, and complies with the 2004 revision of the Declaration of Helsinki. We chose patients with esophagus cancer at tumor stages T3 or T4 or with unresectable lymph nodes as indications of preoperative chemoradiation therapy. Of the patients receiving esophagectomy and excluding those who received preoperative radiotherapy, chemotherapy, or exploratory surgery, a total of 127 patients with esophagus cancer had cervical esophagogastrostomy from March 2008 to September 2010: 79 (62.2 %) men and 48 (37.8 %) women, with a mean age of 59.1 ± 7.0 years. All patients were diagnosed by fibergastroscopic biopsy, with 1 case of small cell carcinoma, 3 cases of adenocarcinoma, and the rest with squamous carcinoma. Barium meal tests, computed tomography (CT) scans, and color Doppler ultrasonography were used to confirm diagnosis. A total of 9 patients had cancer located in the upper thoracic portion, 81 in the middle thoracic portion, and 37 in the lower thoracic portion. The patients were staged according to the tumor, node, metastasis (TNM) Classification of Malignant Tumors, 7th edition (International Union Against Cancer, 2009), with 7 cases staged at IA, 9 at stage IB, 17 at stage IIA, 43 at stage IIB, 27 at stage IIIA, 21 at stage IIIB, and 3 at stage IIIB. Because middle and lower esophageal carcinomas are more common, left thoracotomy with two-field lymphadenectomy was sufficient in most cases to access mediastinal and perigastric lymph nodes. If cervical lymph node metastases were diagnosed before surgery, chemoradiotherapy might have been used as an alternative for similar long-term survival and better quality of life. Diabetes mellitus, heart disease, and chronic obstructive pulmonary disease were present in 6, 10, and 23 patients, respectively. Patient characteristics are listed in Table 1.

**Table 1:** Clinical characteristics and the relation to leakage

Characteristic	Value	Ratio of leakage	*P*
Age, y			0.33
Mean	59.1 ± 7.0	<65, 2.2 %	
Range	38–72	≥65, 5.6 %	
Sex, M/F	79 (62.2 %)/48 (37.8 %)	3.8 %/2.1 %	0.37
Tumor location			0.60
Upper thoracic	9 (7.1 %)	0	
Middle thoracic	81 (63.8 %)	2.5 %	
Lower thoracic	37 (29.1 %)	5.4 %	
Cell type			0.75
Squamous	124 (97.6 %)	3.2 %	
Adenocarcinoma	3 (2.4 %)	0	
Clinical TNM			0.21
IA	7 (5.5 %)	0	
IB	9 (7.1 %)	11.1 %	
IIA	17 (13.4 %)	11.8 %	
IIB	43 (33.9 %)	0	
IIIA	27 (21.2 %)	3.7 %	
IIIB	21 (16.5 %)	0	
IIIC	3 (2.4 %)	0	
Comorbidity		(Without/With)	
Diabetes mellitus	6 (4.7 %)	0/3.3 %	0.65
Heart disease	10 (7.9 %)	10 %/2.6 %	0.20
COPD	21 (16.5 %)	2.9 %/4.3 %	0.72
Surgical approach			0.47
Left esophagectomy	113 (89.0 %)	3.5 %	
Right esophagectomy	14 (11.0 %)	0	
Hospital stay, d			
Mean	18.5 ± 1.9		
Range	10–31		
ICU stay, d			
Mean	2.2 ± 1.0		
Range	1–9		

*TNM* tumor node, metastasis staging system, *COPD* chronic obstructive pulmonary disease, *ICU* intensive care unit

After surgery, the patients received chest CT and upper gastrointestinal X-ray at month 3 and 6. At those same time points, related symptoms, such as dysphagia, heartburn, acid regurgitation, and nocturnal cough, were investigated. Dysphagia was graded according to a published method (0 = no dysphagia, 1 = difficulty swallowing solid food, 2 = able to swallow soft food, 3 = able to swallow liquids, and 4 = unable to swallow food).^9^ The frequency of heartburn, acid regurgitation, and nocturnal cough was graded according to the GerdQ criteria (0 = no related symptoms, 1 = ≥1 day per week, 2 = 2–3 days per week, and 3 = 4–7 days per week).^10^

### Surgical Procedure

**Fig. 1:**
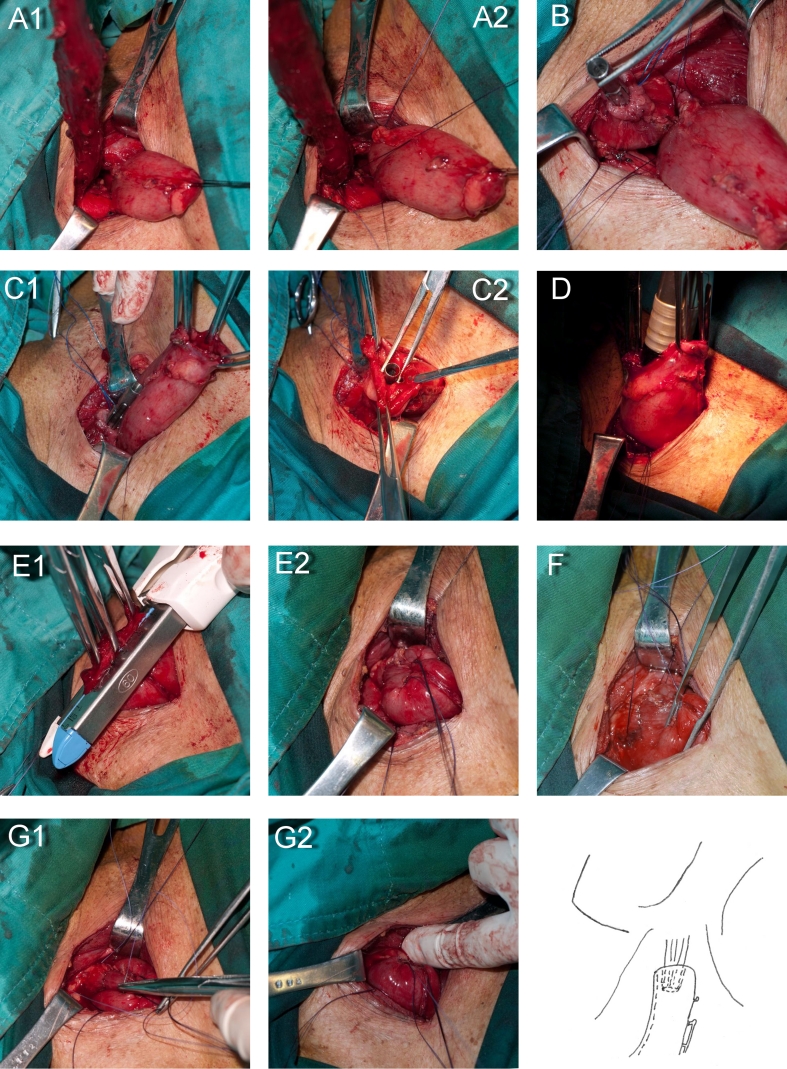
Illustration of circular stapled anastomosis with embedded esophagus. **a** A row of 4-0 sutures was placed in a horizontal mattress fashion between the muscularis of the esophagus and the musculoserosa of the stomach, and the purse stitch of the esophagus was completed using a clamp. **b** The specimen was excised. The anvil was placed into the esophagus and the stitch was tied carefully. **c** A 2.5-cm gastrotomy was made at the top of the gastric conduit, and the rod of the anvil was inserted into the gastric cavity. **d** The anvil was connected to the body of the stapler and fired. **e** The redundant stomach was excised using a linear stapler. **f** The posterior stitches were tied with attention to draw the stomach upward toward the esophagus. This procedure embedded the posterior of the anastomosis into the stomach cavity. A row of 4-0 interrupted sutures in a horizontal mattress fashion was completed over the remaining of circumference of the esophagus and stomach. **g** The anastomosis was fully embedded and the stomach was folded upward around the remaining esophagus

Left thoracotomy was performed on 113 patients, and right thoracotomy with upper midline abdominal incisions was performed on 14 patients that had middle or upper esophageal carcinomas with potential involvement of the trachea and the azygos vein or enlarged upper right mediastinal lymph nodes. After mobilizing the esophagus and stomach, lymphadenectomy of the chest and upper abdomen were routinely performed. The gastric conduit was formed with a linear stapler (Ethicon TLC55; Johnson & Johnson Medical, Shanghai, China), and an incision was made following the anterior border of the sternocleidomastoid muscle. The esophagus was mobilized and brought up to the level of the thyroid cartilage. The gastric conduit was brought out in an orthotopic position, with the lesser curvature facing right and the greater curvature facing left. The anastomotic portion of the esophagus and stomach was scored first. A row of 4-0 sutures was placed in a horizontal mattress fashion between the muscularis of the esophagus (3 cm up from the scored portion) and the musculoserosa of the stomach (2 cm down from the scored portion), and tied until the stapled anastomosis was completed. After the purse stitch of the esophagus was clamped, the specimen was excised. Ethicon SDH25 or SDH21 (Johnson & Johnson Medical, Shanghai, China) were used for the circular stapled anastomosis. An anvil was placed into the esophagus and the stitch was tied carefully. A 2.5-cm gastrotomy was made at the top of the gastric conduit, and the rod of the anvil was pushed into the gastric cavity through the scored portion. The anvil was then connected to the body of the stapler and fired. After checking the integrity of the anastomosis, a nasogastric tube and a nasointestinal tube were inserted. The redundant stomach was excised using a linear stapler. The posterior stitches were tightened to draw the stomach upward toward the esophagus. This procedure embedded the posterior of the anastomosis into the stomach cavity. A row of 4-0 interrupted sutures in a horizontal mattress fashion was completed over the remaining circumference of the esophagus and stomach, about 2 cm from the anastomosis. The anastomosis was then fully embedded and the stomach was folded upward around the remaining esophagus (Fig. 1). A rubber strip at the neck incision and a chest catheter were used for drainage.

### Postoperative Treatment

The drainage catheter and rubber strip were removed 3–4 days later, and enteral nutrition was administered through a nasointestinal tube after 3 days. One week after surgery, the gastric tube was removed and patients began taking food. Anastomotic leakage was considered to be resolved when digestive juice emerged at the incision or, after oral administration of methylene blue, blue liquid flowed out of the incision. The mean length of hospital stay was 18.5 ± 1.9 days and the mean stay in intensive care units was 2.2 ± 1.0 days.

## Results

### Surgical Results

Hand-sewn end-to-side anastomosis was conducted in 2 patients whose stomachs were not long enough for the mechanical procedure. The anastomosis was not embedded in 2 patients because of high tension at the anastomosis site. The remaining patients successfully underwent the complete surgical procedure [123 of 127 (96.9 %)]. In 2 patients, anastomotic bleeding occurred, and after hemostasis was achieved by sewing and binding, the anastomosis was embedded. One patient showed a tear of the esophageal muscularis after gastroesophagostomy, which was embedded after repair.

### Postoperative Complications

The total incidence of complications was 20 of 123 (16.3 %). Anastomotic leakage was seen in 4 patients (3.3 %) on days 4, 6, 8, and 10 after surgery, and after debridement and draining, the leakage stopped within 10–14 days. Other complications included pneumonia in 8 patients (6.5 %), arrhythmia in 5 patients (4.1 %), and injury of the recurrent laryngeal nerve in 3 patients (2.4 %). One patient (0.8 %) died as a result of acute respiratory failure induced by pneumonia. The relationship of leakage and clinical characteristics were tested by Chi-square tests; no special risk factor was found (Table 1).

### Postoperative Review

After surgery, chest CTs and upper gastrointestinal X-rays were reviewed for 123 patients at month 3 and 6, respectively. The results noted that stricture (<1.0 cm) of anastomosis occurred in 12 patients, stricture (<0.5 cm) in 2 patients, and the total incidence of stricture was 14 of 123 (11.4 %). Severe stricture occurred in 2 patients and severe dysphagia occurred in 6 patients (8 of 123; 6.5 %); after gastroscopic biopsy excluded cancer recurrence, these patients received dilatation treatment. A total of 18 dilatation sessions were conducted in all, equaling 2.25 times for each patient over an interval of 2 weeks. Symptoms relevant to gastroesophageal reflux are shown in Table 2. Six months after surgery, the incidence of dysphagia, heartburn, acid regurgitation, and nocturnal cough were 39 of 123 (31.7 %), 62 of 123 (50.4 %), 11 of 123 (8.9 %), and 16 of 123 (13.0 %), respectively.

**Table 2:** Postsurgery symptom scores of dysphagia and gastroesophageal reflux

Symptom	Score							
	3 Months after surgery				6 Months after surgery			
	0	1	2	3	0	1	2	3
Dysphagia^a^	93	26	5	3	88	32	7	0
Heartburn	87	31	9	0	65	44	12	6
Acid regurgitation	115	12	0	0	116	9	2	0
Nocturnal cough	118	8	1	0	111	13	2	1

^a^No patient with dysphagia had grade 4

## Discussion

In recent years, cervical esophagogastrostomy has been performed for many purposes. Two or three fields of lymph node dissection after subtotal esophagectomy could reduce the risk of cancer recurrence.^11^ Cervical esophagogastrostomy is a necessary component of transhiatal esophagectomy or minimal invasive surgery.^12^,^13^ Cervical esophagogastrostomy demands excellent surgical skill and the benefit of experience, due to the limited space at the neck and to protect the recurrent laryngeal nerve while freeing the upper portion of the esophagus. In a meta-analysis of randomized comparative studies, the incidence of postoperative anastomotic leakage and injury of the recurrent laryngeal nerve after cervical esophagogastrostomy were higher than those of intrathoracic anastomosis, but the sample sizes of the studies were small.^14^ For thoracic cancers in the middle and upper thirds of the esophagus, cervical esophagogastrostomy could be an inevitable choice for their radical resection.

The skilled application of anastomosis via circular stapler results in improved safety, comparable or superior to hand-sewn anastomosis.^8^,^15^,^16^ In a comparative study of 26 hand-sewn vs stapled anastomoses, postoperative anastomotic leakage and death shortly after surgery showed conflicting results between the methods, so benefits and drawbacks were unable to be determined.^17^ In recent years, mechanical or semi-mechanical side-to-side anastomosis using a linear stapler has become popular, and the incidence of postoperative anastomotic leakage and anastomotic stricture have been shown to be reduced, but the longer residual end of the esophagus and gastroesophageal reflux remain concerns.^18^–^20^ Toh et al. reported a triangle anastomosis method in which an end-to-end method with good anastomoses between mucosa and blood vessels could reduce the incidence of postoperative anastomotic leakage and anastomotic stricture.^21^ Szücs et al. and Henriques et al. have also reported a lower occurrence of postoperative anastomotic leakage by a telescope-type anastomosis method, which places the remaining esophagus into the stomach cavity.^22^,^23^ However, both studies suffered from complex manipulations and the lack of controls.

At our hospital, we made two improvements based on the conventional stapled anastomosis with circular stapler. First, the anvil penetrated the stomach cavity, connected with the body of the stapler in the stomach cavity, and fired, requiring a shorter gastric conduit and reducing injury caused by pulling the stomach. Second, embedding the remaining esophagus and anastomosis in the stomach cavity facilitates healing and reduces the incidence of gastroesophageal reflux. A prerequisite for successfully performing these procedures is a piece of long- and thick-enough tubular stomach. Otherwise, embedding the anastomosis is hard to complete. Initially, we thought that the diameter of the stapler was irrelevant to postoperative anastomotic leakage and anastomotic stricture, but the appropriate stapler should be selected according to the width of the esophagus.^24^ The anastomoses should be carefully checked before firing to prevent dislocation occurring between mucosa or muscular layers, and appropriate depth should be taken into account. The mobilized esophagus needs to be long enough to both embed and prevent the formation of stomach-esophagus angles after surgery, which could cause dysphagia. The incidence of postoperative anastomotic leakage in this study was 3.3 %; we thought that embedding the anastomosis could reduce the occurrence of postoperative anastomotic leakage.^5^–^8^,^15^,^16^,^18^–^20^

Currently, no recognized standard exists for anastomotic stricture because of the difficulty in evaluating distensibility. In a follow-up study of 9 patients who received semi-mechanical cervical esophagogastrostomy, no patients showed anatomic stricture, all patients experienced reflux laryngitis, and 5 patients showed subjective dysphagia, which was considered to be related to postsurgical dysfunction.^19^ We thought that dysphagia was not only related to the size of anastomosis, but also related to the angle and distensibility of anastomosis, as well as swallowing muscle function. In our study, we considered a <1.0-cm diameter of anastomotic stoma in barium meal tests as criteria: the incidence of stricture was 11.4 % and dilatation was conducted in 6.5 % of patients. As shown by the scoring of dysphagia, medium to severe dysphagia was accounted for 7 of 123 cases (5.7 %). These data were similar to those in the side-to-side anastomosis.^5^,^8^,^18^–^20^

**Fig. 2:**
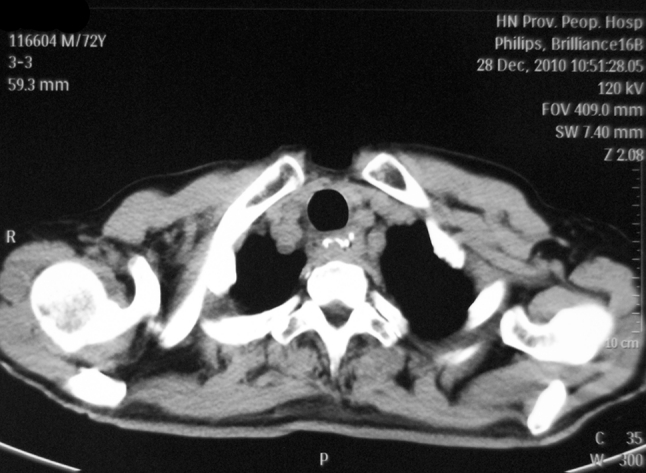
Closed anastomosis at the horizontal position

**Fig. 3:**
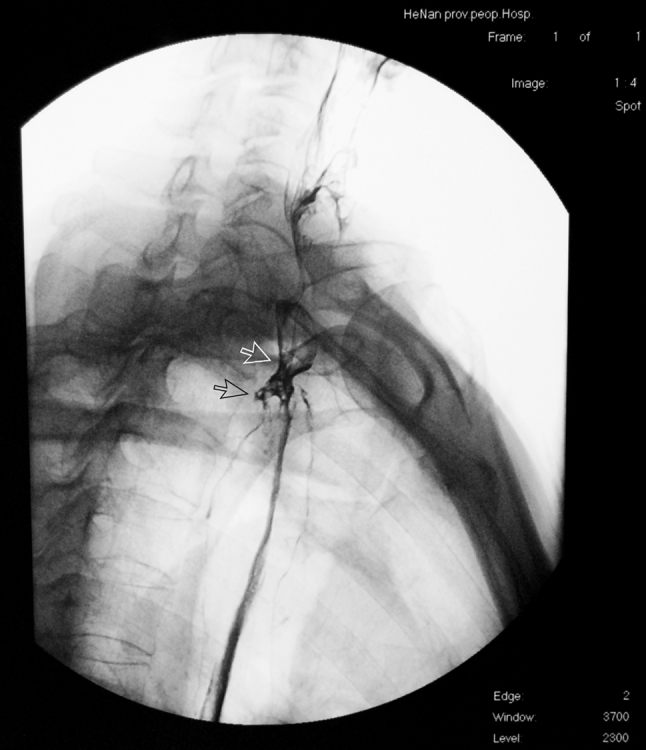
Open anastomosis during a barium meal test (*black arrow*) and compression of the esophagus by the upper portion of the stomach (*white arrow*)

Gastroesophageal reflux is one of the most common postoperative complications.^3^,^25^ We considered that embedding the remaining esophagus not only facilitates the healing of the anastomosis, but also plays an antireflux effect similar to the folding method at the bottom of the stomach via the volume effect in the proximal stomach and compression of remaining esophagus by the multilayer stomach wall. Figure 2 shows the closed anastomosis at the horizontal position. Figure 3 shows an open anastomosis during a barium meal test and the compression of the esophagus by the upper portion of the stomach. Since it was hard to test acid in the remaining esophageal tissue, gastroesophageal reflux was evaluated according to subjective symptoms such as heartburn and acid regurgitation. The incidence of heartburn was 32.5 and 50.4 % in months 3 and 6 after surgery, respectively, which was much higher than that of acid regurgitation (9.8 and 8.9 %, respectively). Although it was thought that the pyloroplasty could reduce the occurrence of reflux, we only dissociated the connective tissue around the pylorus instead of conducting pyloroplasty, and no severe duodenogastric reflux occurred.^26^. We thought that the gastric acid secretion of a nerve-free stomach could gradually recover over time, which was demonstrated by comparing the 3- and 6-month data, while no obvious change in acid regurgitation rate was observed, similar to other published results that indicated that embedding in the proximate stomach presented an antireflux effect.^25^

In conclusion, mechanical anastomosis with a circular stapler is easy and convenient, with just a one-time procedure. Embedding the remaining esophagus not only facilitates the healing of the anastomosis but can also reduce the incidence of gastroesophageal reflux. These results need to be confirmed in randomized controlled studies, and the antireflux effect should be further investigated.
